# Bioproduct engineering: Channeling life science with industrial practice

**DOI:** 10.1002/elsc.202000074

**Published:** 2020-10-26

**Authors:** Guanghui Ma

**Affiliations:** ^1^ Institute of Process Engineering Chinese Academy of Sciences State Key Laboratory of Biochemical Engineering No.1 Bei‐Er‐Jie, Zhong Guan Cun, Haidian District Beijing 100190 P. R. China

The rise of biotechnology has opened up a new paradigm in life sciences. With its rapid development, biotechnology craves for the enhanced industrialization of the high‐end bioproducts to combat against the pandemics, find the cure for the chronic diseases, create cleaner fuels, and increase farming yields, etc. However, there is a tremendous gap between up‐stream biotechnology and down‐stream industrial practice, resulting in the limited translational efficiency for biotechnology.

To address this, State Key Laboratory of Biochemical Engineering, Institute of Process Engineering – Chinese Academy of Sciences (SKLBCE‐IPE‐CAS), along with the pioneers across the world, usher in a new era of bioproduct engineering, which strategically merges biotechnologies with the practical methodologies. From raw materials to high‐end bioproducts, bioproduct engineering covers the innovative strategies and researches in bio‐reaction, bio‐separation and bio‐formulation engineering. In the past 30 years, SKLBCE has strived to make the world better through unraveling the underlying sciences in the industrial bio‐applications, and aimed to cultivate novel amplification and industrialization processes, with high quality, high yield, low cost and environmental‐friendly designs. Along the way, we have established various unique techniques, such as membrane emulsification for drug formulation, solid fermentation, multi‐column protein separation and on‐column protein refolding and translated various works to the industries, such as anticancer delivery systems, vaccine adjuvants, separation media for vaccines and proteins, PEGylated protein/peptide drugs, and so forth.

This special issue, guest edited by Prof. Guanghui Ma, highlights the most innovative multidisciplinary researches in the bioproduct engineering, including bio‐reaction, bio‐separation and bio‐formulation. It is composed of two review‐type articles and nine research articles by the colleagues from SKLBCE‐IPE‐CAS, Tokyo University of Agriculture and Technology, and Islamic Azad University. We hope to introduce novel ideas and knowledge for bioproduct engineering, through the presented articles, which featured the recent progress in bioreaction (bioreactors, biocatalysts), bio‐separation and diagnosis (separation medium, circulating tumor cell detection, Shiga toxin, etc.), as well as the novel formulations for vaccines, biopesticides, functional fragrance, etc.).

We are honored to guest edit this special issue, and thank all authors and referees for their excellent work and unyielding supports. In addition, our gratitude also goes to the *Engineering in Life Sciences* editorial team for their enthusiastic dedications. Contribution from our colleagues at SKLBCE and other cooperating institutes and universities are also sincerely appreciated.

